# Confirmatory Factor Analysis of the Malay Version Comprehensive Feeding Practices Questionnaire Tested among Mothers of Primary School Children in Malaysia

**DOI:** 10.1155/2014/676174

**Published:** 2014-11-04

**Authors:** Shamarina Shohaimi, Wong Yoke Wei, Zalilah Mohd Shariff

**Affiliations:** ^1^Department of Biology, Faculty of Science, Universiti Putra Malaysia, 43400 Serdang, Selangor, Malaysia; ^2^Department of Nutrition and Dietetics, Faculty of Medicine & Health Sciences, Universiti Putra Malaysia, Serdang, Malaysia

## Abstract

Comprehensive feeding practices questionnaire (CFPQ) is an instrument specifically developed to evaluate parental feeding practices. It has been confirmed among children in America and applied to populations in France, Norway, and New Zealand. In order to extend the application of CFPQ, we conducted a factor structure validation of the translated version of CFPQ (CFPQ-M) using confirmatory factor analysis among mothers of primary school children (*N* = 397) in Malaysia. Several items were modified for cultural adaptation. Of 49 items, 39 items with loading factors >0.40 were retained in the final model. The confirmatory factor analysis revealed that the final model (twelve-factor model with 39 items and 2 error covariances) displayed the best fit for our sample (Chi-square = 1147; *df* = 634; *P* < 0.05; CFI = 0.900; RMSEA = 0.045; SRMR = 0.0058). The instrument with some modifications was confirmed among mothers of school children in Malaysia. The present study extends the usability of the CFPQ and enables researchers and parents to better understand the relationships between parental feeding practices and related problems such as childhood obesity.

## 1. Introduction

The issue of obesity and its negative health and psychological consequences has been emphasized for decades [1]. The understanding of the various factors that cause or relate to the problem of obesity is undoubtedly important especially now with the prevalence of obesity increasing sharply in developing countries including Malaysia. The Third National Health and Morbidity Survey (NHMS III) showed the prevalence of overweight for children aged < 18 years was 5.36%. Among them, children in the age group of 7–9 reported the highest prevalence, that is, 6.8% [2] which was higher than the 4.4% prevalence reported in 1996 [3]. Therefore, there is a need for research and improved public health preventive programs, which should begin from childhood in order to prevent the development of obesity and the subsequent health problems in adulthood.

Previous research suggests that parental feeding practices are related to child's weight. For example, parental restriction on child's eating was positively related to child's BMI [4–6]. The possible explanation for this relationship was parental restriction on particular foods might draw attention of their children to that restricted foods and increase the children's desire to consume the restricted foods [7, 8]. Higher consumption of restricted foods such as high-fat foods and sweets may result in higher weight gain. However, evidence on the relationship between feeding practices and child's weight has been inconsistent. Some studies revealed no relationship between parental restriction and child's weight [9, 10]. However, Faith et al. [11] reported that parental extreme restriction in allotting child food choice resulted in children with lower BMI. Different levels of restriction may have different influence on development of childhood obesity. Parents are more likely to encourage or pressure thinner children to eat more [6, 12, 13]. However, pressuring to eat can be ineffective in promoting intake of a food; in fact, it may instead lower the intake and create negative affective responses to the food [12]. If children refuse to eat healthy foods that they are encouraged to eat, but instead love to eat unhealthy foods that they are discouraged to eat, this may result in weight gain. Too much control in child food intake has been found to be positively related to child's higher intake of high-fat foods and snacks [14]. However, in cross-sectional studies of the association between parental feeding practices and child's weight status, it is not possible to determine if parental feeding practice causes an increase or decrease in child's weight or has an effect on child eating. While parental feeding practices may influence child's weight and eating, there is also the possibility that child eating and weight also influence parenting [15].

Most researches on parental feeding practices were conducted using questionnaires as an instrument to examine the relationship between maternal feeding practices and child's eating behaviour or child's weight status [5, 15, 16]. Child feeding questionnaire (CFQ) is the most commonly used instrument to assess the relationship. It measures three aspects of parental feeding practices (restriction, pressure to eat, and monitoring) in addition to their perceptions and concerns about the development of obesity proneness among their children [17]. These three mostly examined feeding practices might not be sufficient to give a complete picture of the parental feeding practices. Furthermore, some other potential feeding practices which might influence the development of childhood obesity could not be measured. A more comprehensive and relatively new instrument called comprehensive feeding practices questionnaire (CFPQ) was developed [18]. CFPQ covers a wider range of behaviours that are related to the feeding practices. The developers of CFPQ have conducted three studies attempting to capture more comprehensive behaviours that parents have regarding feeding their young children aged 18 months to 8 years.

CFPQ should be considered and applied in research on parental feeding practices because it covers not only restriction, monitoring, and pressure to eat from the CFQ but also factors from preschooler feeding questionnaire (PFQ; [19]) including child control, emotion regulation, and food as a reward. In addition, the developers have added some new items and new factors including encouraging balance and variety, healthy environment, modelling, and teaching about nutrition through a thorough literature review, whilst involvement of child was a factor suggested by parents [18]. Restriction was categorized into two subscales: restriction for health and restriction for weight control (adapted from Dutch Eating Behaviour Questionnaire) which could give clearer purposes for the restriction. The developers tested the validity and reliability of the 12-factor feeding practices instrument among American parents of children who were mostly Caucasian (>90%). The final 12-subscale model for the samples showed a good fit with *χ* ^2^(1061) = 1580, RMSEA = 0.057, and CFI = 0.98.

A recent validation study of the Norwegian version of the CFPQ was carried out among parents of adolescents aged 10–12 years. This early phase study tested the validity of 42 items (out of 49 items) using principal component analysis (PCA). In a 10-factor solution, a few items did not load on the expected factor, while items of the same scales split into different factors. In addition, there was one item with a low factor loading which is below 0.4. However, the expected correlation between the CFPQ subscales and the attitude scales (parents' concern and feelings of responsibility) and the acceptable internal consistency reliability of the subscales made the CFPQ a valid tool for measuring parental feeding practices [20].

While most of the CFPQ validation studies involved small, homogenous samples, Haszard et al. [21] conducted a CFPQ validation study on a large, diverse sample of 1013 children in New Zealand aged between 4 and 8 years. This study found that the original 12-factor model was not a good fit and instead conducted exploratory factor analysis which resulted in a five-factor model which consisted of healthy eating guidance, monitoring, parent pressure, restriction, and child control [21].

In order to examine the suitability of the existing instrument among the target population, the fit of the factor model could be tested through confirmatory factor analysis (CFA). CFA is used to test the factor structure or model which has been predetermined by the researchers. A set of indices will be used to evaluate the fit of the measurement model [22].

The availability of a valid and reliable instrument to assess parental feeding practices is very important to our understanding of the factors that influence the development of overweight or obesity during childhood. However, to date, studies on the validity of the structural model of the CFPQ are limited. Nothing is known about the validity and reliability of CFPQ among Asian population including the Malaysian population. The objective of the article was to test the suitability of the Malay version of CFPQ among mothers of primary school children by applying confirmatory factor analysis to evaluate the factor structure. We also evaluated the validity and reliability of the factor model which best fit the sample.

## 2. Methods

### 2.1. Sample Recruitment and Size

The participants of the present study were mothers and their children were aged 7–9 years studying in primary schools located in the state of Selangor and Federal Territories of Kuala Lumpur and Putrajaya, which has the highest prevalence of childhood obesity according to the NHMS III [2]. Recommendation for sample size estimation for CFA is based on subject-to-item (or observed variable) ratio. According to Hair et al. [22], the minimum sample size for factor analysis is at least a ratio of 5 : 1 (observation: number of items). Since the assessed instrument contained 49 items, the estimated minimum sample size was 245. In order to overcome the problems of unwillingness of subjects to participate and questionnaires not fit to use because too many unanswered questions, at least 20% more subjects were recruited. Thus, the minimum number of dyads (mothers and their children) to be recruited was 294.

This was a proportionate random sampling study in which the sample was recruited according to the ratio of Malaysia's three main ethnicities, Malay, Chinese, and Indian (6 : 3 : 1). Malay, Chinese, and Indian students were mostly sampled from the national schools (*Sekolah Kebangsaan*, SK), Chinese national-type schools (*Sekolah Jenis Kebangsaan Cina*, SJK (C)), and Tamil national-type school (*Sekolah Jenis Kebangsaan Tamil*, SJK (T)), located in urban areas in the state of Selangor and Federal Territories of Kuala Lumpur and Putrajaya. The names of the schools were drawn from three different boxes for the different types of school. The name of a school was drawn every time after a school had been visited. This was done until the requirements were fulfilled (the minimum sample size and the ratio of the main ethnicities).

First, we made appointments with the randomly selected schools and distributed the questionnaire and parental consent forms to the children of the randomly selected class. The second visit was made after a week to collect the questionnaires and parental consent forms from the children. The height and weight of children with given consent were measured. In the present study, 1310 questionnaires have been distributed and 603 of them were returned with consent letter. We excluded dyads with children who were not given consent by parents (*n* = 100), children whose height and weight were not measured (*n* = 73), children who were not living together with their mothers (*n* = 2), children whose mothers did not report their socio-demographic information (*n* = 2), children of other ethnicities (*n* = 6), and outliers (with extreme anthropometric measurements or identified by the Mahalanobis distance test; *n* = 23). We found no significant differences between our included and excluded dyads on potentially important demographic variables such as ethnicity and child's gender. Finally, we had a total of 397 dyads that were eligible for the statistical analyses.

### 2.2. Measures

#### 2.2.1. Malay Version Comprehensive Feeding Practices Questionnaire (CFPQ-M)

The original CFPQ was translated into Malay Language. The instrument contained 12 scales with a total of 49 items that measured feeding practices using a 5-point Likert scale. The response scales were anchored by the terms “never,” “rarely,” “sometimes,” “mostly,” and “always” for items numbered 1 to 13, while “disagree,” “slightly disagree,” “neutral,” “slightly agree,” and “agree” for items numbered 14 to 49. Some items were modified for cultural adaptation. For example, “how much do you keep track of the snack food (potato chips, Doritos, cheese puffs) that your child eats?” was modified to “How much do you keep track of the snack food (potato chips, fish cracker, Twisties, Mamee) that your child eats?” (see the appendix).

#### 2.2.2. Sociodemographic Information

Mother and child's sociodemographic information such as their date of birth, weight, height, and ethnicity were obtained using the questionnaire. Other pieces of information asked included mother's educational level, number of children, occupation, individual and household income, and employment of maid as well as child's gender.

#### 2.2.3. Anthropometric Measurement

Mothers self-reported their height and weight. Child's height and weight were measured by trained staff during the second visit to the school. Shoes were taken off and height was determined by a fixed SECA height measuring device model M132 and recorded to the nearest 0.1 cm. Weight was determined by a TANITA digital bathroom scale model HD-308 and recorded to the nearest 0.5 kg. Weight status of the mothers was determined using the classification of body mass index (BMI) defined by WHO [23]. Obesity was defined by BMI >30.0, while overweight was defined by BMI 25.0–30.0. For children, their weight status was determined by age- and sex-specific *z*-score of BMI according to the definition by WHO growth reference [24].

### 2.3. Translation

We adopted and simplified the TRAPD framework, that is, translation, review, adjudication, pretesting, and documentation [25] to translate the English version of the CFPQ into Malay. This method was chosen because it is inexpensive and fits the short time frame. The original English version CFPQ was translated into Malay by our research group. The translated version was examined by a translator from the Faculty of Modern Languages and Communication, Universiti Putra Malaysia. His task was also to identify problematic items and deciding whether to retain the original translation or make changes to it. The translation aimed to fulfil content equivalence which means each item's content is relevant in the target culture [26]. Therefore, some items were modified in order to fit the culture in Malaysia. For example (Item 2 and Item 16), common snack foods consumed by children in Malaysia are different from those eaten by children in Western countries. Pretesting of the translated CFPQ was done among 40 parents of primary school children in order to collect their comments about the understanding of the items. We reviewed and discussed each item until a general agreement was reached. There was one modified item. “I withhold sweets/dessert from my child in response to bad behaviour” was changed to “I withhold sweets/dessert from my child in response to bad behaviour such as being lazy and talking back to parents” for a better understanding. Satisfaction of the layout of the questionnaire was also taken into account.

### 2.4. Test-Retest

One week test-retest reliability of the instrument was evaluated among a small number of parents (*n* = 45). The parents answered the CFPQ-M twice and similar or same mean scores were expected to be obtained from the two occasions.

### 2.5. Statistical Analyses

SPSS version 17.0 was used in the statistical analyses. The normality of the distribution of the score was first estimated by using skewness and kurtosis values. The values fall in the range of −1 to 1 indicating the scores were normally distributed [27]. Descriptive statistics was used to describe the mean score for each subscale. Confirmatory factor analyses (CFA) were conducted using AMOS version 18.0. We specified the hypothesized model [18] and examined the factorial validity of the model in our sample. Four indices were chosen according to the strong recommendations by Kline [28]: Chi-square (*χ* ^2^), comparative fit index (CFI), root mean square error of approximation (RMSEA), and standard root mean square (SRMR). Criteria to define a good model for this study were *χ* ^2^ to degree of freedom ≈ 2 : 1 [29], CFI ≥ 0.90 [30], RMSEA ≤ 0.05 [31], and SRMR < 0.08 [32]. Models were revised until a good-fit-model was obtained. Items with factor loadings of less than 0.40 were subject to elimination [33]. Modification indices were examined in order to improve the fit of the model. In addition, the internal consistencies of the subscales of the final model were examined using Cronbach's alpha to measure the reliability, that is, the consistency of response to items of scale over the content or time. Cronbach's alpha was used instead of other reliability measures because it is widely reported and acceptable despite providing a lower bound of reliability. Correlations between subscales were calculated using Pearson's correlation. Correlations between subscales were examined to check for overlapping of factors. Overlapping of factors is indicated by high correlation that equals or is more than 0.85 [34]. The usual retest interval ranged from 2 days to 14 days which is not too long to change things or not too short until the participants remember their first response [35]. One week test-retest reliability of the instrument was estimated using paired sample *t*-test [36]. The instrument was considered test-retest reliable if all of the mean differences of the two mean scores from the two occasions are not statistically different (*P* < 0.05).

## 3. Results

### 3.1. Child and Mother Characteristics

The final sample was comprised of 397 observations for confirmatory factor analysis. Hundred percent of the recruited mothers (mean age 38.06 ± 4.73) and their children (8.23 ± 0.95) lived together. The samples were made up of 53% of Malay, 35% of Chinese, and 12% of Indian which was similar to the ratio of Malaysia's three main ethnicities, Malay, Chinese, and Indian (6 : 3 : 1). The nutritional status of most of the mothers and children was normal (mean BMI = 23.08 ± 4.15 and 16.83 ± 3.48 kg/m^2^ resp.). Girls accounted for 56.9% of the children.

### 3.2. Distribution of Score

Mean scores, standard deviation, skewness, and kurtosis values of the twelve subscales are presented in Table 1. The values of skewness and kurtosis showed that the scores of the subscales were normally distributed.

### 3.3. Fit of the Models

#### 3.3.1. Hypothesized Model

The hypothesized model with 12 factors and 49 items suggested by Musher-Eizenman & Holub [18] showed a poor fit to our sample (CFI < 0.90 and SRMR was not available in the output). Moreover, this model contained 9 items with loadings of <0.40, that is, 2 environment items (Item 16 “I keep a lot of snack food (potato chips, Doritos, cheese puffs) in my house” and Item 37 “I keep a lot of sweets (candy, ice cream, cake, pies, pastries) in my house”), 2 restrictions for health purposes items (Item 40 “I have to be sure that my child does not eat too much of his/her favourite foods” and 43 “I have to be sure that my child does not eat too many sweets (candy, ice cream, cake, or pastries)), 1 food as a reward item (Item 36 “I withhold sweets/dessert from my child in response to bad behaviour), 1 restriction for weight control item (Item 18 “I have to be sure that my child does not eat too many high-fat foods), 1 pressure to eat item (Item 17 “My child should always eat all of the food on his/her plate”), 1 teaching about nutrition item (Item 42 “I tell my child what to eat and what not to eat without explanation.”), and 1 child control item (Item 12 “Do you allow this child to leave the table when s/he is full, even if your family is not done eating?”), suggesting that removal of these items would improve the model fit.

*Model 1.* Model 1 was formed when all of the 9 low loading items were eliminated from the hypothesized model. This model contained 40 items. It met all the chosen cut-offs except CFI. Therefore, the model was revised.

*Model 2 and Model 3.* Based on the modification indices, error covariances were included one by one forming Model 2 (between Item 34 and Item 35) and Model 3 (between Item 34 and Item 35; Item 24 and Item 38) in order to improve the fit of the models. Compared to Model 1, Model 2 and Model 3 had better fit (the CFI values were higher) although they still did not meet all the criteria of a good-fit model.

*Model 4.* A low-loading item, Item 38, was found in Model 3. It was eliminated to form Model 4. The error covariance between Item 24 and Item 38 was dropped because of the exclusion of Item 38. The CFI value was still below the chosen cut-off.

*Model 5.* Based on the modification indices, another error covariance was added between Item 27 and Item 39 for the model improvement. Model 5 was accepted as the final and best model, *χ* ^2^/*df* = 1.809, CFI = 0.900, SRMR = 0.058, and RMSEA = 0.045. The results of the goodness-of-fit indices of the hypothesized and tested models are shown in Table 2. (For the excluded items, please refer to the appendix).

### 3.4. Description of the Final Model

**Figure 1:**
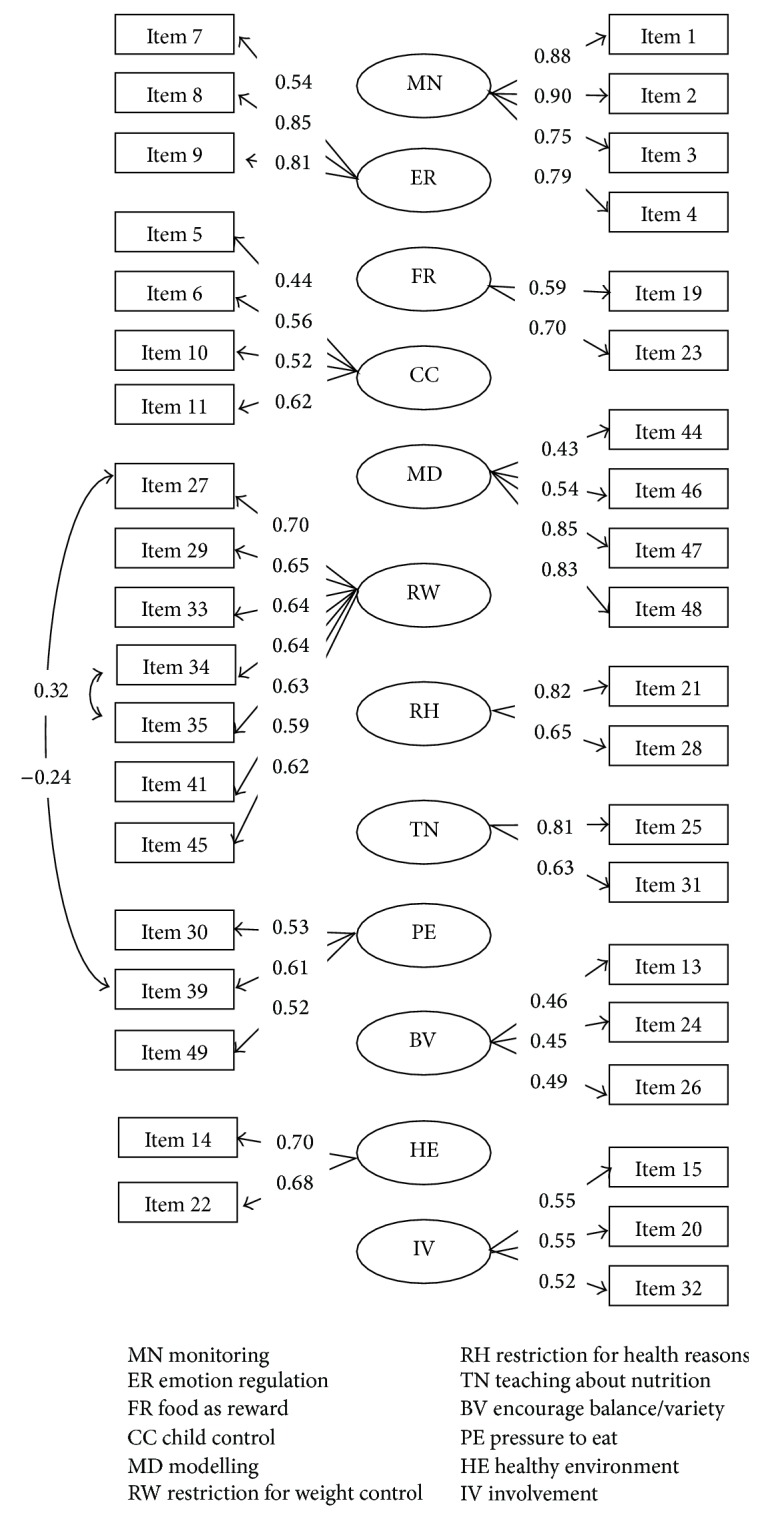
Factor loadings and error covariances of the final model of CFPQ-M.

The final model of CFPQ-M is shown in Figure 1. The final 12-factor model contained 39 items, with factor loadings ranging from 0.43 to 0.90, and two error covariances. All of the loadings were with *P* < 0.001 which indicated that the 39 items were meaningful to the responding factors. Face validity also indicated that all of the items were measuring the respective factors.

Correlations between the subscales of the final model were examined (Table 3). Low correlations (*r* < 0.8) indicated that there was no overlapping of the subscales. The highest correlations (*r* = 0.42) were found between teaching about nutrition and modelling and involvement of child, respectively, as well as healthy environment and encouragement for balance and variety. Mothers who reported higher scores in teaching about nutrition were also more likely to encourage balance and variety and prepare healthy environment (*r* = 0.32, resp.) but were less likely to let their children control over his or her eating. Mothers who reported higher pressure to eat were less likely to monitor their children's eating and restrict their children's eating for weight control (*r* = −0.16 and −0.21, resp.). Healthy environment, involvement of child, and modelling were positively correlated with each other (*r* = 0.32–0.36). Food as a reward and emotion regulation also showed positive relationship (*r* = 0.32).

The instrument had acceptable test-retest reliability with all the mean scores of subscales obtained from two occasions showing no significant differences (Table 4). Internal consistency reliability of the subscales was examined (Table 5). All of the subscales showed acceptable reliability with *α* ≥ 0.60, except for encouragement for balance and variety (*α* = 0.45), involvement (*α* = 0.55), pressure to eat (*α* = 0.57), and food as a reward (*α* = 0.59).

The correlation between the mean scores of the feeding practices and child's BMI were examined (Table 5). Restriction for weight control and pressure to eat correlated with child's BMI (*r* = 0.38 and −0.30, resp., *P* < 0.01). Besides that, monitoring and food as a reward also correlated with child's BMI (*r* = 0.10 and −0.13, *P* < 0.05).

## 4. Discussion

CFPQ was developed by Musher-Eizenman & Holub [18] to assess parental feeding practices comprehensively. The present study is the first study to assess the validity and reliability of CFPQ across the major ethnic groups in Malaysia. This is important as there is a need for validation of the instrument in a multiethnic population. This is because different cultures have different developmental approaches to eating behaviour which involve learning and experience and development of food preferences in childhood [37]. It is very important to have an instrument which is suitable for the target sample before having further investigations using the instrument.

The confirmatory factor analysis revealed that the final model (twelve-factor model with 39 items and 2 error covariances) displayed the best fit for our sample. The loadings of all items in the final model were above 0.4 indicating acceptable validity of the overall model [33]. A total of 10 low-loading items were eliminated. Item 12 is about allowing child to leave the eating table after he or she is full, even if the family members were not done eating yet. Encouraging the child to remain seated at the dining table after eating is done may be considered education of table manners. Parents were advised to educate and encourage their school-aged child to do so and wait for the others to finish eating [38]. However, allowing the child to leave the eating table was probably to avoid the child disturbing others from eating. This shows that it may not be table manners. This reason would probably explain the low factor loading of Item 12.

Item 16 is about keeping a lot of snack food in the house, while Item 37 is about keeping a lot of sweets in the house. A lot of mothers disagreed with keeping a lot of snack food as well as sweets. The child would consume more snack food and sweets instead of high-fat foods if snack food and sweets were prepared at home. Musher-Eizenman and Holub [18] claimed that there were no sufficient strong items to define the subscale of providing healthy environment and thus collected opinions from parents for items development. However, more research should be carried out to develop questions which could correctly measure the healthy environment subscale.

Item 17 “My child should always eat all of the food on his/her plate”, an item from pressure to eat subscale, was eliminated. We found that most of the mothers agreed with Item 17 but less agreed to get their children to eat anyway when their children was not hungry, ate only small helping, or even finished eating (three other items in the subscale). A possible explanation for this situation is that mothers always encouraged their children to finish all the food (with suitable portion) ready for them. According to Jain et al. [39], mothers knew how much their children would eat and they did not agree to make the child to eat more.

Item 18 is about making sure that child does not eat too many high-fat foods. From the results, we could see that the mothers were relatively more pronounced in restricting the child from eating high-fat foods (Item 18) rather than eating foods that might make the child fat or eating less (all items except Item 18 in the restriction for weight control subscale). Undoubtedly, most people know that high-fat foods contribute most to the total fat as well as energy intake. For example, a fast food meal accounted for between 47.5% and 93.5% of a daily fat consumption guideline for adults [40]. In other words, children most probably will overconsume fat in a day if one of the daily meals is a fast food meal. In fact, reducing intake of high-fat foods which contain saturated fat,* trans*-fat, and cholesterol is for maintenance of good health and prevention of chronic diseases [41]. Classifying Item 18 into restriction for weight control subscale is questionable.

Item 36 “I withhold sweets/dessert from my child in response to bad behaviour” might not be measuring food as a reward subscale in our sample. Our results revealed that mothers offered their children favourite foods as a reward for doing something good but they disagreed to withhold favourite foods as a punishment for the children's bad behaviours. As a result, Item 36 loaded poorly onto the food as a reward subscale and was thus removed. Our food as a reward subscale content (Item 19 “I offer my child his/her favorite foods in exchange for good behaviour” and Item 23 “I offer sweets (candy, ice cream, cake, pastries) to my child as a reward for good behaviour” in the present study) were similar to those of Corsini et al. [42]. In their study, the food as a reward subscale which was solely defined by the two items was superior in terms of internal consistency reliability and overall model fit.

The items in the restriction for health reasons subscale, Item 40 “I have to be sure that my child does not eat too much of his/her favorite foods” and Item 43 “I have to be sure that my child does not eat too many sweets (candy, ice cream, cake, or pastries), are about the need for making sure that the child does not eat too much of his or her favourite foods and sweets, respectively. The factor loadings of Item 40 and Item 43 (factor loadings <0.3) were much more lower compared to the other two items in this subscale (about regulating child's eating so that he or she would not overeat his or her favourite foods or junk foods with factor loadings ≈0.7).

The two subscales of restriction with distinct purposes were introduced by Musher-Eizenman and Holub [18]. Besides weight control and health purposes, there may be some other reasons for restriction. Item 43 concerns sweets which include candy, ice-cream, and cake, which are usually consumed by children. Mothers might be more sensitive to sweets as sweets are known as a cause for tooth decay and this could be the motivation for the restrictive action. Restriction of various foods could also be due to dietary belief and information given by healthcare givers [43]. However, the reason for low loading of Item 40 was not clear. Therefore, further research is required to examine the motivation behind the restrictive action.

Item 42 “I tell my child what to eat and what not to eat without explanation” is a negative item, which is about telling a child what to eat and what not to eat without explanation. Removal of items including Item 42 (factor loading = 0.16) improved the fit of the model. The possible reason could be that some mothers persuaded their child to consume healthy and nutritious foods but did not really explain the reasons for not allowing the child to eat or not eat any kind of food.

Item 38 is about encouraging child to eat a variety of foods. It was the last item to be removed in the stage of model respecification. Its factor loading was about the acceptable level. However, the removal of the item improved the fit of the model. Item 38 is the only item regarding promoting the consumption of varied foods, while the rest of the items in this subscale are about promoting the consumption of healthy foods and new foods.

The existence of overlapping of measurement errors could be due to small and unmeasured common variables. Two added error covariances improved the fit of model as suggested by the modification indices. Errors of measurements are expected to be unique and uncorrelated [44] but this statement is not realistic in practice [45]. Modification indices suggested a positive error covariance to be added between Item 34 and Item 35 (both from the subscale of restriction for weight control). Item 34 and Item 35 are concerning the foods eaten by child that will make him or her fat. Meanwhile, a negative error covariance was to be added between Item 27 and Item 39 for the model fit improvement. This finding was interpreted as indicating the bipolar nature of the two items. Item 27 and Item 39 were about encouraging the child to eat less and trying to get the child to eat more, respectively. Correlations between subscales of the final factor model were examined using Pearson's correlation. There was no overlapping of factors. CFPQ subscales correlated in the theoretical expected way. For example, restriction for weight control was negatively correlated with pressure to eat more. Mothers who used food as a reward were more likely to use food to regulate child's emotion status. These two subscales were grouped as “nonnutritive uses of food” [4].

In the present study, the internal consistency reliability of the encouraging for balance and variety subscale in CFPQ-M was the lowest (*α* = 0.45) among all of the subscales, which was similar to that of the study by Musher-Eizenman and Holub [18]. Low internal consistency suggests that the items do not correlate well together [46]. The definition for this subscale should be reexamined in order to improve the internal consistency of the subscale. Other subscales with Cronbach's alpha of less than 0.6 were involvement, pressure to eat, and food as a reward. These four subscales showed borderline internal consistency reliability in the present study. This may be due to the insufficient number of items, especially food as reward subscale which consisted of two items. Adding the number of items will improve the Cronbach's alpha [47]. Nevertheless, reliability of the instrument was supported by an alternate form of reliability test, that is, test-retest reliability, which showed the mean scores of each subscale were stable over time.

We examined the relationships between feeding practices and child's BMIs. Higher restrictions for weight control and monitoring were significantly correlated with heavier children. Meanwhile, higher food as a reward and pressure to eat were associated with lower child's BMI. These results were similar to the results from previous studies which suggested that parents probably encouraged the thinner children to eat more, while overweight or obese children were controlled from overeating by parents [6, 48, 49]. The relationships between parent and child could be bidirectional [50] meaning that maternal feeding practices can be shaped or influenced by child's nutritional status.

The strength of this study is that there was sufficient number of dyads of mother-child to conduct factor analyses. In addition, the ratio of the sample approximated the ratio of Malaysia's three main ethnicities and thus may extend the generalizability of the results to the Malaysian population.

A limitation of our study was insufficient number of items to define a subscale. In our final model of CFPQ-M, there were four subscales containing only two items. A subscale defined by two items can be considered stable when there is high correlation between items but low correlation with other items [34]. Despite four subscales containing only two items, we chose to retain the subscales because the present study is an early stage validation study of CFPQ-M. Further studies are required as more than two items are generally recommended to measure a distinct subscale [51].

We believe that the current study gives relevant contribution to research on parental feeding practices and child's weight status. It helps to widen the application of CFPQ to other populations. Through the present study, we showed that the CFPQ-M is a potential instrument to investigate parental feeding practices among Malaysian. This particular interest was aroused due to the increasing prevalence of childhood obesity in Malaysia. We suggest qualitative research such as semi-structured interviews to be carried out to investigate the interpretation of each item by mothers for improvement of items and further conceptual development of parental feeding practices instrument. We stress the need for replication of factor studies. If similar factor structure is yielded, the confidence of the factor structure revealed in our study and the generalizability of the results would increase. Our study makes a good start of using CFPQ in Malaysia. A better understanding of the maternal feeding practices would then allow more research or interventions to deal with the identified problems such as childhood obesity which stem from particular feeding practices.

## Figures and Tables

**Table 1:** Mean score, standard deviation, skewness, and kurtosis for CFPQ-M subscales.

	Mean ± S.D.	Skewness	Kurtosis
Monitoring	3.36 ± 0.86	0.17	−0.67
Emotion regulation	2.04 ± 0.75	0.58	0.07
Food as reward	2.72 ± 1.12	0.17	−0.70
Child control	2.57 ± 0.64	0.18	0.04
Modelling	4.12 ± 0.76	−0.69	−0.21
Restriction for weight control	3.34 ± 0.95	−0.26	−0.51
Restriction for health reasons	3.86 ± 1.20	−0.87	−0.26
Teaching about nutrition	4.24 ± 0.80	−1.03	0.88
Encourage balance/variety	4.02 ± 0.74	−0.72	0.06
Pressure to eat	2.89 ± 1.00	−0.02	−0.66
Healthy environment	4.25 ± 0.83	−0.96	0.18
Involvement	3.88 ± 0.84	−0.65	0.09

**Table 2:** Goodness-of-fit indices for the 12-factor models.

Model (12-factor)	*χ*² (df)	*χ* ^2^/df	CFI	SRMR	RMSEA (90% CI)
Hypothesized model [18]	2363.8 (1061)^*^	2.228	**0.765**	NA	0.056 (0.053–0.059)
Model 1: 40 items	1299.7 (674)^*^	1.928	**0.868**	0.060	0.048 (0.044–0.052)
Model 2: 40 items, 1 error covariance	1258.0 (673)^*^	1.869	**0.877**	0.060	0.047 (0.043–0.051)
Model 3: 40 items, 2 error covariances	1236.0 (672)^*^	1.839	**0.881**	0.059	0.046 (0.042–0.050)
Model 4: 39 items, 1 error covariance	1160.1 (635)^*^	1.827	**0.887**	0.059	0.046 (0.042–0.050)
Model 5: 39 items, 2 error covariances	1146.8 (634)^*^	1.809	0.900	0.058	0.045 (0.041–0.049)

Note: value below chosen cut-off is in bold.

*χ*² (df):Chi-square statistics (degree of freedom); CFI: comparative fit index; SRMR: standard root mean square residual; RMSEA: root mean square error of approximation; CI: confidence interval.

^*^ *P* < 0.001.

**Table 3:** Correlations between subscales of the final model of CFPQ-M.

	(1)	(2)	(3)	(4)	(5)	(6)	(7)	(8)	(9)	(10)	(11)	(12)
(1) MN	1.00											
(2) ER	−0.02	1.00										
(3) FR	−0.00	0.32^**^	1.00									
(4) CC	0.12^*^	0.28^**^	0.15^**^	1.00								
(5) MD	0.24^**^	0.00	0.07	−0.03	1.00							
(6) RW	0.12^*^	0.03	−0.02	−0.01	0.22^**^	1.00						
(7) RH	0.22^**^	0.12^*^	0.14^**^	0.24^**^	0.17^**^	0.18^**^	1.00					
(8) TN	0.18^**^	−0.06	−0.00	−0.14^**^	0.42^**^	0.23^**^	0.09	1.00				
(9) BV	0.31^**^	0.08	0.12^*^	0.11^*^	0.30^**^	0.15^**^	0.09	0.32^**^	1.00			
(10) PE	−0.16^**^	0.28^**^	0.26^**^	0.06	0.05	−0.21^**^	0.02	0.00	0.05^**^	1.00		
(11) HE	0.25^**^	−0.05	0.07	−0.07	0.34^**^	0.10^*^	0.18^**^	0.32^**^	0.42^**^	−0.01	1.00	
(12) IV	0.18^**^	−0.03	0.04	0.03	0.32^**^	0.24^**^	0.05	0.42^**^	0.34	−0.04	0.36^**^	1.00

^*^ *P* < 0.05, ^**^ *P* < 0.01.

**Table 4:** One week test-retest reliability of the final model of CFPQ-M subscales (*n* = 45).

Feeding practices	Mean ± S.D.		Mean difference	*t*	*P*
	T1	T2			
Monitoring	3.73 ± 0.66	3.83 ± 0.65	−0.10	−0.66	0.516
Emotion regulation	1.91 ± 0.68	1.95 ± 0.58	−0.04	−0.30	0.764
Food as reward	2.71 ± 1.20	2.74 ± 1.01	−0.29	−0.12	0.903
Child control	2.74 ± 0.51	2.65 ± 0.49	0.09	0.86	0.398
Modelling	4.13 ± 0.87	4.25 ± 0.83	−0.13	−1.05	0.300
Restriction for weight control	3.58 ± 0.93	3.57 ± 0.91	0.01	0.60	0.952
Restriction for health reasons	4.26 ± 1.05	4.14 ± 0.99	0.12	0.57	0.574
Teaching about nutrition	4.03 ± 1.02	4.12 ± 0.79	−0.09	−0.67	0.511
Encourage balance/variety	4.08 ± 0.65	4.05 ± 0.68	0.02	0.15	0.882
Pressure to eat	2.66 ± 1.01	2.86 ± 0.96	−0.20	−1.18	0.248
Healthy environment	4.16 ± 0.75	4.00 ± 0.82	0.16	1.14	0.264
Involvement	3.96 ± 0.86	4.10 ± 0.66	−0.15	−1.26	0.218

**Table 5:** Internal consistency reliability (*α*) of the final model of CFPQ-M subscales and their correlations (*r*) with child's BMI.

	*α*	Child's BMI (*r*)
Monitoring	0.90	0.10^*^
Emotion regulation	0.76	−0.05
Food as reward	0.59	−0.13^*^
Child control	0.61	<−0.01
Modelling	0.73	0.04
Restriction for weight control	0.83	0.38^**^
Restriction for health reasons	0.69	0.06
Teaching about nutrition	0.67	0.01
Encourage balance/variety	0.45	0.01
Pressure to eat	0.57	−0.30^**^
Healthy environment	0.64	−0.04
Involvement	0.55	0.04

^*^ *P* < 0.05; ^**^ *P* < 0.01.

## Appendix

### (CFPQ-M)

*Child Control.* Parents allow the child control of his/her eating behaviors and parent-child feeding interactions.

(5) Do you let your child eat whatever s/he wants?
(6) At dinner, do you let this child choose the foods s/he wants from what is served?
(10) If this child does not like what is being served, do you make something else?
(11) Do you allow this child to eat snacks whenever s/he wants?

*Emotion Regulation.* Parents use food to regulate the child’s emotional states.

(7) When this child gets fussy, is giving him/her something to eat or drink the* first *thing you do?
(8) Do you give this child something to eat or drink if s/he is bored even if you think s/he is not hungry?
(9) Do you give this child something to eat or drink if s/he is upset even if you think s/he is not hungry?

*Encourage Balance and Variety.* Parents promote well-balanced food intake, including the consumption of varied foods and healthy food choices.

(13) Do you encourage this child to eat healthy foods before unhealthy ones?
(24) I encourage my child to try new foods.
(26) I tell my child that healthy food tastes good.
(38) I encourage my child to eat a variety of foods.

*Environment.* Parents make healthy foods available in the home.

(14) Most of the food I keep in the house is healthy.
(16) I keep a lot of snack food (potato chips, Doritos, and cheese puffs) in my house.** R**
(22) A variety of healthy foods are available to my child at each meal served at home.
(37) I keep a lot of sweets (candy, ice cream, cake, pies, and pastries) in my house.** R**

*Food as Reward.* Parents use food as a reward for child behavior.

(23) I offer sweets (candy, ice cream, cake, and pastries) to my child as a reward for good behavior.
(36) I withhold sweets/dessert from my child in response to bad behavior.
(19) I offer my child his/her favorite foods in exchange for good behavior.

*Involvement.* Parents encourage child’s involvement in meal planning and preparation.

(15) I involve my child in planning family meals.
(20) I allow my child to help prepare family meals.
(32) I encourage my child to participate in grocery shopping.

*Modeling.* Parents actively demonstrate healthy eating for the child.

(44) I model healthy eating for my child by eating healthy foods myself.
(46) I try to eat healthy foods in front of my child, even if they are not my favorite.
(47) I try to show enthusiasm about eating healthy foods.
(48) I show my child how much I enjoy eating healthy foods.

*Monitoring.* Parents keep track of child’s intake of less healthy foods.

(1) How much do you keep track of the sweets (candy, ice cream, cake, pies, and pastries) that your child eats?
(2) How much do you keep track of the snack food (potato chips, Doritos, and cheese puffs) that your child eats?
(3) How much do you keep track of the high-fat foods that your child eats?
(4) How much do you keep track of the sugary drinks (soda/pop, kool-aid) this child drinks?

*Pressure.* Parents pressure the child to consume more food at meals.

(17) My child should always eat all of the food on his/her plate.
(30) If my child says “I’m not hungry,” I try to get him/her to eat anyway.
(39) If my child eats only a small helping, I try to get him/her to eat more.
(49) When he/she says he/she finished eating, I try to get my child to eat one more (two more, etc.) bites of food.

*Restriction for Health.* Parents control the child’s food intake with the purpose of limiting less healthy foods and sweets.

(21) If I did not guide or regulate my child’s eating, s/he would eat too much of his/her favourite foods.
(28) If I did not guide or regulate my child’s eating, he/she would eat too many junk foods.
(40) I have to be sure that my child does not eat too much of his/her favorite foods.
(43) I have to be sure that my child does not eat too many sweets (candy, ice cream, cake, or pastries).

*Restriction for Weight Control.* Parents control the child’s food intake with the purpose of decreasing or maintaining the child’s weight.

(18) I have to be sure that my child does not eat too many high-fat foods.
(27) I encourage my child to eat less so that he/she will not get fat.
(29) I give my child small helpings at meals to control his/her weight.
(33) If my child eats more than usual at one meal, I try to restrict his/her eating at the next meal.
(34) I restrict the food my child eats that might make him/her fat.
(35) There are certain foods my child should not eat because they will make him/her fat.
(41) I do not allow my child to eat between meals because I do not want him/her to get fat.
(45) I often put my child on a diet to control his/her weight.

*Teaching about Nutrition.* Parents use explicit didactic techniques to encourage the consumption of healthy foods.

(25) I discuss with my child why it is important to eat healthy foods.
(31) I discuss with my child the nutritional value of foods.
(42) I tell my child what to eat and what not to eat without explanation.** R**

*Note.* Factor names are presented with a brief operational definition of the factor content. Item numbers indicate the order in which they were presented in the survey. Items numbered 1–13 utilize a 5-point response scale “never, rarely, sometimes, mostly, and always.” Items numbered 14–49 utilize a 5-point scale with different anchors, “disagree, slightly disagree, neutral, slightly agree, and agree.” Items marked with an **R** were reverse coded. In the final model, the eliminated items due to low loadings included items numbered 12, 16, 17, 18, 36, 37, 38, 40, 42, and 43.
